# Magnesium and Zinc in Schizophrenia

**DOI:** 10.3390/biomedicines13092249

**Published:** 2025-09-12

**Authors:** Mihai Nechifor

**Affiliations:** Department of Pharmacology, Gr. T Popa University of Medicine and Pharmacy, 700115 Iasi, Romania; mihainechifor@umfiasi.ro; Tel.: +40-7-4450-8642

**Keywords:** schizophrenia, magnesium, zinc, BDNF, neuroinflammation, oxidative stress

## Abstract

Schizophrenia is a severe recurrent chronic disease that affects a large number of patients. Numerous genetic and epigenetic factors are involved in the pathogenesis of this disease. The involvement of magnesium and zinc has been relatively little studied and often underestimated. The main mechanisms by which zinc and magnesium are involved in the pathogenesis of these diseases is their influence on the neurotransmitter systems at the cerebral level (dopaminergic, glutamatergic, serotoninergic, GABAergic, catecholaminergic and cannabinoid systems). The action of many other factors involved in one form or another in the pathogenesis of schizophrenia is influenced by magnesium and zinc. Among these factors, we mention neuroinflammation, oxidative stress, nuclear factor kB (NF-kappaB), galanin, brain-derived neurotrophic factor (BDNF), substance P(SP), oxytocin, ACTH, prolactin and others. There are also data related to some interactions between antipsychotic medication and the two cations, as well as to disturbed physiological processes (sleep, appetite) in patients with schizophrenia. The existing data show that the concentrations of the two cations must always be determined and the deficits immediately corrected.

## 1. Introduction

Schizophrenia is one of the most severe human chronic diseases. This psychosis is a multifactorial, severe recurrent disease during which the patient partially loses contact with reality. Schizophrenia is associated with a shorter life by 8–15 years compared to the general population [1,2]. Magnesium and zinc have many actions in the human body. These are essential elements and important biometals for our body. Since the pathogenesis of schizophrenia involves important dysfunctions in synaptic transmission, the release of neurotransmitters and the activity of the receptors on which they act, the involvement of disturbances in zinc and magnesium concentrations in the pathogenesis of this disease is a problem. The pathogenic mechanisms of schizophrenia are not completely understood today, but there is substantial evidence supporting a complex involvement of magnesium and zinc in the pathogenesis of this severe disease.

### 1.1. Magnesium

There are about 600 magnesium-dependent enzymes [3]. Zinc is a co-factor of more than 300 enzymes and 2000 transcription factors [4]. At the level of the central nervous system, these two bivalent cations influence the synthesis of numerous neurotransmitters and their action in the brain. Both zinc and magnesium are essential biometals in the human body [5]. Magnesium is a predominantly intracellular cation. Of the total amount of magnesium in the body, 99% is found in the cells.

The serum magnesium concentration in normal adults is between 1.82 and 2.30 mg/dL (0.75–0.95 mmol/L) [6,7]. As magnesium readily crosses the blood–brain barrier, the extracellular concentration of magnesium in the brain is similar to that in serum [8]. The concentration of magnesium in the cerebrospinal fluid in healthy adults is 1.25 ± 0.14 mmol/L [9].

### 1.2. Zinc

Zinc is located both extracellularly and intracellularly. The muscles, bones and brain are the organs where the most zinc is found. The concentration of zinc in the brain is about 8–10 times higher than in other organs [10].

In the brain, about 20% of the total amount of zinc is found in free extracellular form. A big part of free zinc in the brain is localized in the glutamatergic terminals from the hippocampus, frontal cortex and amygdala [11,12]. The concentration of zinc in the brain is 150 micromol/L. This concentration in the brain is about 10 times higher than the serum concentration of this cation [13,14]. This cation is a neuromodulator at the level of many synapses in the brain [15].

The existing data regarding the concentrations of zinc and magnesium in patients with schizophrenia are heterogeneous and sometimes contradictory, but many results show low levels of the two cations in these patients. We believe that the heterogeneity of the existing results regarding magnesium concentrations has several causes: the age of the disease is often not indicated and it is difficult to establish the actual onset of schizophrenia; in some studies the treatments prior to determining the magnesium concentrations are not indicated; for treatments that could influence this concentration, some research studies do not mention whether or not food supplements containing magnesium and other cations were used.

Some authors found a low plasma level of magnesium in schizophrenic patients before treatment [16,17]. There are data showing that the risk of schizophrenia is significantly higher in people with low levels of plasma zinc and magnesium [18].

Other authors found no differences in serum magnesium concentrations in drug-free patients compared to healthy people [19]. There are data that show that the level of magnesium in the brains of schizophrenic patients post-mortem is not different from the concentration of this cation in the control group, without a correlation with the duration of the period without neuroleptic treatment before death [20]. In other studies, the concentrations of magnesium in the CSF and in plasma were higher in schizophrenics than in normal people [21,22], along with a reduced magnesium concentration in acute schizophrenics versus remitted patients [23]. Data on zinc concentrations in patients with schizophrenia are less heterogeneous than those on magnesium.

In drug-naïve patient with schizophrenia, the level of plasma zinc was reduced compared to a control group (normal peoples) [24]. The zinc concentration in scalp hair of schizophrenics is reduced compared to normal people [25]. Some authors support the theory that zinc deficiency is very important or essential in the pathogenesis of schizophrenia. It is also argued that gestational zinc deficiency is also important in the etiopathogenesis of this disease [26,27].

Not only are the concentrations of magnesium and zinc in schizophrenic patients important but also the ratios between the concentrations of these cations and those of other biometals. The ratio between zinc and copper concentrations is altered in schizophrenics. The copper level is higher in these patients compared to healthy control subjects [28].

An important problem is the heterogeneity of schizophrenia and the fact that over time the diagnostic criteria of this disease have varied [29]. The lifetime prevalence range of schizophrenia in the world is about 0.4–0.7%, with significant variations between different geographical areas [30]. The life expectancy in patients with this disease is reduced by 20%. Genetic and epigenetic factors are involved in the etiopathogenesis of schizophrenia.

The most important brain systems involved in the pathogenesis of schizophrenia include the dopaminergic system, serotonergic system, glutamatergic system, GABAergic system, cholinergic system and cannabinoid system. Oxidative stress and inflammation are also involved [31]. Based on the existing data regarding the numerous implications of magnesium and zinc in the activity of the central nervous system, this work aims to show the importance of the involvement of the two divalent cations in the pathogenesis of schizophrenia.

Magnesium and zinc influence the synthesis and synaptic release of neurotransmitters, their transport and their action at the receptor level.

## 2. The Factors Involved in the Pathogenesis of Schizophrenia

### 2.1. Dopaminergic System

The dopaminergic theory of the pathogenesis of schizophrenia claims that the hyperfunction of the cerebral dopaminergic systems is the most important mechanism for the pathogenesis of this disease [32].

Dysregulation of dopaminergic neurotransmission is considered a key mechanism of schizophrenia. There are multiple interactions between the dopaminergic system and the noradrenergic, serotonergic and glutamatergic systems in the pathogenesis of this disease.

An increased synthesis of dopamine and an increased stimulation of dopaminergic receptors is considered important in the pathogenic mechanism of this disease [33,34]. There is also a genetic involvement in the pathogenesis of schizophrenia. Among the genes identified as modified in schizophrenia is the gene corresponding to D2-like dopamine receptors [35]. D2-like receptors are not the only dopaminergic receptors involved in the pathogenesis of schizophrenia. The density of D4 receptors is increased by about six times in these patients [36].

Studies conducted with positron emission tomography have shown that in patients with schizophrenia, the levels of dopamine in certain brain areas such as the striatum and the substantia nigra are increased. Additionally, the activity of tyrosine hydroxylase, the enzyme involved in the synthesis of catecholamines, is elevated [37]. Dopamine synthesis is also elevated in the brains of individuals with prodromal symptoms of schizophrenia, which strongly implicates dopamine in the pathogenesis of this disease [38]. However, the dysfunction of the cerebral dopaminergic system only partially explains the pathogenesis of schizophrenia. The effectiveness of some antipsychotics is positively correlated with their antagonistic effect at the level of D2 and D3 receptors [39]. Antipsychotics that block D2 receptors reduce the positive symptoms of the disease but have little or no influence on the negative symptoms of the disease [40].

The activity of the dopamine transporter protein is regulated by zinc. This cation could block the transport of dopamine (both inward and outward) through a non-competitive mechanism [41]. In experimental studies on rats, zinc significantly increased the concentrations of dopamine in some areas of the brain. Additionally, this cation amplified the effect of insulin at the brain level [42].

Magnesium reduces striatal dopamine release under certain stressful conditions such as anoxia [15], as well as the release of brain dopamine stimulated by calcium ions and by other substances. Addictive substances such as nicotine, cocaine and morphine also produce significant stimulation of dopamine release in some brain regions [43,44]. Dopamine release is also increased by some endogenous substances that do not cause addiction, such as endothelins [45].

The absence of magnesium in the superfusion buffer in microdialysis studies resulted in doubling of basal dopamine release [46]. Experimental magnesium deprivation (by a magnesium restricted diet) causes a significant increase in the concentrations of dopamine and 5-hydroxyindole-3-acetic acid in the brain [47]. Magnesium at a dose of 1.2 mM reduced, in experimental studies, the NMDA-induced release of dopamine in the striatum [48,49]. The strong stimulating effect of presynaptic dopamine release by glutamate occurs only at low concentrations or in the absence of magnesium ions. These data show that maintaining a normal magnesium concentration reduces excessive activity of dopaminergic systems involved in the pathogenesis of schizophrenia [50].

Adenosine is another important substance for CNS function. It reduces the release of dopamine from various brain areas. The inhibitory effect of adenosine is potentiated by magnesium [51]. This is another mechanism by which it reduces some of the manifestations of schizophrenia due to increased activity of dopaminergic systems.

Stress or at least some forms of stress is considered a contributing factor involved in the development of schizophrenia. The interaction between the D2 receptor and the disrupted in schizophrenia 1 (DISC1) protein is implicated in producing psychotic effects, and its experimental inhibition in animals reduces these effects [52], but no action of magnesium or zinc on this protein is detected.

### 2.2. Glutamatergic System

The glutamatergic system is also involved in the schizophrenia pathogenesis. The involvement of glutamatergic system dysfunctions in the pathogenesis of schizophrenia is considered a possible explanation, especially for the negative symptoms of the disease. The hypothesis of the major involvement of the glutamatergic system in the pathogenesis of schizophrenia was proposed based on the observation that phencyclidine, a glutamate antagonist at the N-methyl-d-aspartate (NMDA) receptor level, produces in normal people some of the positive and negative symptoms encountered in schizophrenic patients [53].

The glutamate concentration is increased in the brains of schizophrenics before treatment [54]. Due to the hypofunction of NMDA receptors in these patients, this increased level of glutamate acts strongly on non-NMDA receptors. This fact would cause the appearance of some symptoms of schizophrenia [55]. The administration of ketamine (a non-competitive blocker of NMDA receptors) leads to an increase in the cortical concentration of glutamate simultaneously with the appearance of symptoms resembling certain manifestations of schizophrenia [56]. Zinc is mainly localized at the level of glutamatergic synapses. This biometal acts both at the level of synthesis and release of glutamate in the brain and at the level of the main brain receptors for glutamate, the NMDA and metabotropic glutamate receptors. Zinc is an allosteric antagonist of NMDA receptors.

At the level of NMDA receptors, there is a site for glycine that has a modulatory role. Agonists of this site increase the activity of NMDA receptors [57,58]. It is not clear what role this glycine site plays in the pathogenesis of schizophrenia. Memantine, which is a non-competitive antagonist of NMDA receptors for glutamate, also ameliorates some negative symptoms of schizophrenia.

Zinc also acts on the level of α-amino-3-hydroxy-5-methyl-4-isoxazolepropionic acid (AMPA) receptors. At low concentrations (30–300 microg/mL), zinc potentiates the activity of these receptors, but at high (over normal) concentrations (1 mM/mL) it inhibits the activity of AMPA receptors [59,60]. Some of the zinc ions enter in neurons through calcium-permeable AMPA/kainate receptors. There are complex functional relationships between NMDA receptors and AMPA glutamate receptors. While NMDA receptor activation has slower kinetics and is primarily involved in generating long-term synaptic potentiation, AMPA receptors are important for the rapid transmission of the biological signal given by glutamate at the synaptic level. The functional balance between these two receptor categories is essential for the functioning of the brain’s glutamatergic systems. AMPA receptors are also involved in neuroplasticity [60]. Although the exact role of AMPA receptors in the pathogenesis of schizophrenia is not known, it is important to note that the functioning of both types of glutamate receptors is influenced by zinc ions [61].

There are data showing that allosteric modulators of mGlu2 receptors reduce the hallucinogenic action of substances such as (-)2,5-dimethoxy-4-bromoamphetamine. These modulators potentiate the action of glutamate on metabotropic glutamate (mGlu2) receptors.

The mGlu3 receptors are located at the level of astrocytes and presynaptically. These receptors are involved in glutamate release inhibition. The mGlu2-3 receptors of glutamate are also important for the release of dopamine and are implicated in the pathogenesis of schizophrenia. The stimulation of these receptors reduces the dopamine release induced by various substances such as amphetamine. Some authors believe that the activation of these receptors could be a new way to treat schizophrenia [62]. Zinc reduces the increase in extracellular glutamate concentration produced by some factors [63]. Influencing the concentration of glutamate in the brain could be a mechanism by which zinc is involved in the evolution of schizophrenia, in the activity of mGlu2-3 receptors of glutamate and in the symptomatology of this disease.

There are studies implicating changes in the SLC30A3 gene, which encodes the ZnT3 zinc transporter in the etiopathogenesis of schizophrenia [64]. ZnT3 is involved in the modulatory effect of zinc on the activity of cerebral glutamatergic systems. Changes in the function and structure of this transporter may be involved in increasing glutamatergic activity in patients with schizophrenia.

At the neuronal level, zinc is primarily stored in presynaptic vesicles and is released in the synapses, where it has a role as a neuromodulator. This cation binds to the GluN2A subunit of NMDA receptors and reduces their activity. However, zinc also has a modulatory role at the AMPA receptor level of glutamate. Zinc released from presynaptic vesicles inhibits the activity of AMPA receptors [65,66]. Zinc depletion of neurons induces their death [6].

Excess zinc is also toxic at the neuronal level, particularly in schizophrenic patients. Decreased concentrations of zinc in the sera and brains of patients with schizophrenia has been repeatedly reported. There are no studies showing an increased concentration of zinc in the brains of healthy individuals with schizophrenia compared to normal subjects.

Magnesium has a very important regulatory role in the activity of NMDA receptors. This cation blocks the calcium entry in the cell by blocking calcium channels coupled with NMDA receptors [67,68]. Magnesium ions interact with NMDA receptors at three different sites.

The first site of action is at the level of the calcium channel. Here, magnesium binds covalently to asparagine. This binding results in the voltage-dependent block of the calcium channel.

The second site of interaction of magnesium with these receptors is at the N-terminal domain of the GluN2B subunit. The third magnesium action site of NMDA receptors is in the same region as the zinc action site [69]. The action of magnesium on NMDA receptors is closely related to the structure of these receptors. Studies have shown that replacing a single amino acid in the GluN2A subunit of the NMDA receptor reduced the Mg^2+^ block at the calcium channel [70]. In clinical practice, due to the major role of magnesium in regulating NMDA receptor activity, the hypomagnesemia that occurs in chronic alcoholism could have implications for the functioning of these receptors and the evolution of patients with schizophrenia.

### 2.3. Serotonergic System

The cerebral serotonergic system is also involved in the pathogenesis of schizophrenia. The platelet serotonin level is higher in schizophrenics. Probably the most important implication is the interaction between the 5-hydroxytriptamine2A receptor (5-HT2AR) and metabotropic glutamate 2 receptor (mGluR2). The two receptors form heteromeric complexes at the neuronal level [71]. This means that any stimulation or blocking of one of the receptors can influence the functioning of the other. The main argument for the involvement of cerebral serotonergic systems in the pathogenesis of schizophrenia is the observation that different hallucinogenic substances stimulate 5HT2 receptors [72,73].

The hallucinations caused by these substances are similar to those of some forms of schizophrenia [74]. An experimental model that exactly reproduces schizophrenia in humans does not exist; therefore, it is difficult to evaluate the involvement of each system of active endogenous substances in the pathogenesis of schizophrenia [75]. Some studies claim that serotonin has an action of reinforcing the activity of the cerebral dopaminergic systems [76]. Antagonists of 5-HT2 receptors have an antipsychotic effect on negative symptoms of schizophrenia [77]. Both divalent cations (magnesium and zinc) influence the activity of the cerebral serotonergic systems but in different ways.

Zinc is co-localized in some regions of the brain (hypothalamus, frontal cortex) with 5-HT(1A) receptors. An important action of zinc is the allosteric modulatory effect at the level of 5HT(1A) receptors [78]. If the concentration of this biometal is 5 micromoles or higher, Zn^2+^ inhibits the binding of agonists to 5-HT(1A) receptors. Unlike zinc, MgCl_2_ reduced 5-HT concentrations in the nucleus accumbens and in the prefrontal area.

### 2.4. GABAergic System

Existing data show that GABA interneurons play an essential role in maintaining a balance between the actions of excitatory systems (predominantly glutamatergic) and inhibitory systems in the brain [79]. Disruption of this balance plays an important role in the pathogenesis of schizophrenia. The functional deficit of the GABAergic systems in some areas of the brain (the frontal cortex) is involved in the pathogenesis of this disease.

The cerebral GABAergic system, especially in the cortex, is affected in patients with schizophrenia. Studies performed using proton magnetic resonance spectroscopy in the frontal cortex in patients with schizophrenia showed reductions in the levels of GABA [80]. A decrease in the expression of GABA genes was revealed by post-mortem studies in the brains of schizophrenics. GABA (B) receptors are located presynaptically at the level of dopaminergic nerve endings. Their stimulation determines the reduction in dopamine release [81]. Not only is GABA synthesis reduced in these patients but also the activity of GABA is transporters affected. Some authors claim that the primary sites of deficiencies of cortical GABAergic systems found in schizophrenia are formed by GABAergic interneurons [82].

This seems to be the key element of the involvement of the GABA system in the pathogenesis of schizophrenia. Any hypoactivity of these receptors causes dopaminergic hyperactivity and the stimulation of glutamate release and activity. Post-mortem studies have shown that the numbers of GABA (B) receptors in healthy people with schizophrenia are reduced in some areas of the brain, such as the temporal cortex and the entorhinal cortex [83]. The reduction in GABAergic system activity is also involved in the disruption of working memory in schizophrenics.

At a concentration of 10 mM of magnesium, the binding of GABA receptor agonists to GABA receptors (A) is increased [84]. The production of the anxiolytic effect of magnesium is mediated by the stimulation of GABA (A) and benzodiazepine receptors. The administration of flumazenil (a competitive antagonist of benzodiazepine receptors, which are localized on the same receptor protein as the GABA (A) receptors) determines the important reduction in the anxiolytic effect of magnesium [85]. Benzodiazepines are positive modulators of the activity of GABA (A) receptors. By stimulating the activity of benzodiazepine receptors, magnesium causes an increase in the activity of cerebral GABAergic systems. Magnesium also potentiates the action of these benzodiazepines through direct action on GABA (A) receptors, and some authors believe that there are magnesium binding sites at the level of these receptors [84]. Experimental studies have shown that presynaptic GABA release under the action of substances such as kainate and quisqualate, which increase this release, is modulated by the synaptic concentration of magnesium [86].

Stimulation by GABA of neuronal development through stimulation of GABA receptors (A) is dependent on magnesium concentration [87]. The stimulation of GABA (A) receptors by their natural agonist also determines the mobilization of intracellular magnesium. Magnesium ions stimulate cyclic AMP response-binding protein (CREB) and mammalian target of rapamycin (mTOR) activity, which is important for neuronal maturation [88]. Both of these factors are translational factors involved in essential cellular processes at the neuronal level (cellular proliferation, cellular response to insulin, energy metabolism and others).

Zinc is also involved in GABA secretion and activity. In zinc-deprived rats, the extracellular zinc levels are low in the amygdala and hippocampus. GABA extracellular concentration decreases in these animals [89]. Unlike magnesium, zinc reduces the activity of GABA (A) receptors by binding to a subunit of these receptors [90].

### 2.5. Cannabinoid System

Experimental data demonstrated a complex involvement of cannabinoids in the production of psychotic symptoms. Delta-9-tetrahydrocannabinol (THC) induces psychotic manifestations in some situations, but cannabidiol has an antipsychotic effect [91]. Experimental studies have shown that magnesium deficiency causes an aggravation of THC neurotoxicity in rats and an increase in animal aggression [92]. The hyperaggressiveness of rats is greater even in cases of a reduced deficiency in magnesium and increases directly proportionally to the severity of this deficiency. Stimulation of presynaptic cannabinoid receptors by their agonists inhibits glutamate release [93]. The activation of presynaptic cannabinoid receptors causes a reduction in the activity of voltage-gated Ca^2+^ channels. A reduced concentration of magnesium increases the entry of calcium into the cell and the intracellular concentration of calcium. In such cases, magnesium acts oppositely to cannabinoid receptor agonists [94]. THC inhibits Mg-ATP-ase from synaptosomes isolated from the rat cerebral cortex. In rat hippocampal neurons in culture, the administration of THC led to glutamatergic transmission by stimulating cannabinoid 1 (CB1) receptors [95].

### 2.6. Cholinergic System

The implications of the cerebral cholinergic system are still not completely understood. A reduction in [(3)H]pirenzepine binding to M1/M4 muscarinic receptors in the anterior cingulate cortex was observed in schizophrenic patients [96]. The dysfunctions of this system are considered to play an important role in the schizophrenia pathogenesis. In post-mortem studies, the binding of [(3)H]pirenzepine in the anterior cingulate cortex to M1 receptors of schizophrenia patients was significantly reduced (by 24% in the superficial cortex and by 35% in the deep cortex) compared to non-schizophrenic people [97]. The activation of muscarinic receptors is important for neuroplasticity [98].

Recent data show that stimulation of cerebral muscarinic receptors with xanomeline reduced psychotic symptoms in Alzheimer’s patients and improved cognition [99]. The psychotic symptoms of these patients share many similarities with those of schizophrenia, without knowing if they always share the same mechanism of production. Nicotinic receptors are also involved in the pathogenesis of schizophrenia, at least in terms of negative symptoms. Administration of alpha-7 nicotinic receptor agonists has shown improvements in negative symptoms of schizophrenia [100]. Smoking seems to have the same effect. Magnesium blocks the ion channel coupled with the nicotinic acetylcholine receptors. Intracellular and extracellular magnesium ions exhibit this effect, but the effect of intracellular magnesium is stronger [101]. N-methyl-D-aspartate stimulating the NMDA receptors of glutamate causes 35–100% increases in the release of acetylcholine in the striatum [102]. This increase is inhibited by magnesium (1.2 mM of Mg^2+^), which also has a direct action on muscarinic receptors. Experimental data indicate that Mg^2+^ binds to an allosteric site at the level of M2 receptors. The influence of this binding on the action of acetylcholine in patients with schizophrenia is not known [103]. Mg^2+^ deficiency affects M-cholinergic and serotonergic neurotransmission in the brain [104]. Mg^2+^ (1.2–9.3 mM) increases the acetylcholine release from rat cortex slices [105]. The implications of magnesium in the pathogenesis of schizophrenia are shown in Table 1.

Unlike magnesium, zinc influences the activity of some nicotinic receptors.

Zn^2+^ (100 microM) potentiates the action of nicotine on neuronal alpha4beta4 nACh receptors [112]. A dose of 200 microM of zinc increased the currents elicited by acetylcholine (ACh currents) by 361% for alpha4beta4 and by 182% for alpha1beta1gammadelta nAChRs. 

### 2.7. Neuroplasticity

The decrease in normal neuroplasticity was found in all forms of schizophrenia and is considered an important element in the pathogenesis of the disease [113]. Some authors consider schizophrenia a neuroplasticity disease [114]. The volumes in the hippocampus, neocortex, parahippocampal gyrus and other cerebral structures are reduced in schizophrenia [115]. Cortical atrophy has been demonstrated in numerous cases of schizophrenia [116]. It indicates an increase in neurodegeneration, a reduction in neuroplasticity and a decrease in neurogenesis. Increasing neuroplasticity is considered to be an objective in the treatment of patients with this disease [117].

Both magnesium and zinc increase neuroplasticity. Zinc deficiency inhibits cell proliferation. At a low level of this biometal, the cell cycle stops in the G0/G1 phase [118]. Zinc reduces apoptosis through a double mechanism: it inhibits the activity of caspase 3 (the essential protein in the production of apoptosis) and also has an important role in the transduction of the biological signal of growth factors through action at the level of tyrosine kinase receptors [119].

The disrupted-in-schizophrenia 1 (DISC1) gene is an important gene in schizophrenia and bipolar disorders, and also plays a role in neuroplasticity. This is a multifunctional protein that has numerous interactions with different structures (especially intracellular proteins). DISC1-binding zinc-finger protein (DBZ) is considered to be important for DISC1 action. A lack of zinc can indirectly reduce neuroplasticity in this way. The growth of neurites in the brain is especially dependent on DISC1 [120]. Magnesium also reduces apoptosis and stimulates brain stem cell neurogenesis.

### 2.8. Neuroinflammation

Neuroinflammation is involved in schizophrenia pathogenesis. The levels of proinflammatory cytokines (Il-1 beta, Il-2, IL-6, IL-8, tumor necrosis factor (TNF)) are high in patients with this disease [121]. These cytokines are also involved in neurodegeneration [122]. The increases in the synthesis of pro-inflammatory cytokines and neuroinflammation are also involved in the increase in blood–brain barrier (BBB) permeability in patients with schizophrenia [123]. Some studies have shown that in patients with a high level of proinflammatory cytokines, there are language disorders [124] and a reduction in volume of Broca’s area [125].

The severity of these pathological changes is correlated with the levels of cytokines in the brain and blood of patients with schizophrenia [125]. During the first psychotic episode in young patients (up to 18 years old), an imbalance between pro-inflammatory and anti-inflammatory factors was found, with a clear higher level in the concentration of pro-inflammatory factors [126].

The increased activity of cerebral microglia also plays a role in the pathogenesis of schizophrenia. Activated microglia increase the formation of free radicals and cytokines, but also increase the synthesis of nitric oxide. Microglia is, thus, involved both in the onset and relapses of schizophrenia [127]. Chronic stress is one of the factors that increases the activity of microglia. Experimental studies have shown that stress is one of the factors that increases the synthesis of pro-inflammatory cytokines in the brain [128]. A high level of proinflammatory cytokines is positively corelated with a reduction in cortical grey matter volume in schizophrenic patients [129].

A way through which zinc could have a beneficial effect on patients with schizophrenia is by reducing the synthesis of pro-inflammatory cytokines and cerebral neuroinflammation. The increases in the level of these cytokines both in the early stages of life and later are considered by some authors as a factor involved in the etiopathogenesis of schizophrenia [130]. Zinc and magnesium reduce the synthesis of these pro-inflammatory factors and could improve the evolution of this disease. We believe that the earlier the administration of zinc and magnesium, the more important the reduction in the level of pro-inflammatory cytokines would be before they majorly disrupt the functioning of some cerebral neurotransmitter systems. There are studies that show that increases in the activity levels of anti-inflammatory factors such as nuclear factor-erythroid-2-related factor 2 (Nrf2) and neuronal receptor tyrosine kinase-2 (TrkB), substances that reduce neuroinflammation, could reduce the risk of juvenile prodromal syndromes making the transition to schizophrenia [108].

### 2.9. Oxidative Stress

Oxidative stress is also involved in the pathogenesis schizophrenia. An increased level of oxidative stress and an important disturbance in redox mechanisms has been observed in patients with schizophrenia before treatment [131,132]. Some authors consider oxidative stress to be an essential element in the pathogenesis of this disease [133]. Several mechanisms are known by which oxidative stress is involved in the pathogenesis of schizophrenia.

A reduced activity of antioxidant systems in schizophrenics was observed in the first psychotic episode. The concentration of reduced glutathione is low, as is the total antioxidant status (TAS) [134,135,136]. Reduced glutathione (GSH) is an important antioxidant factor. In schizophrenia, the presence of a polymorphism of the gene for glutathione-S-transferases and a reduction in GSH synthesis have been found [137]. This polymorphism is considered an important risk factor for schizophrenia. In schizophrenia patients, the level of superoxide dismutase (SOD) was lower compared to normal controls, while the concentration of malondialdehyde (MDA) was higher [138]. The reactive oxygen species (ROS) level is also higher. An increased concentration of ROS causes a reduction in the activity of the endoplasmic reticulum pumps followed by an increase in the intracellular concentration of calcium [139]. This has two important consequences: it modifies the release of certain synaptic neurotransmitters (a release dependent on the concentration of calcium) and it disrupts the activity of NMDA receptors for which calcium ions are essential. In patients with schizophrenia, the level of GSH is low in various areas of the brain and especially in the cerebral cortex. The high level of ROS and the low concentration of GSH determine the decrease in GABA production [140]. Consequently, there is a decrease in the activity of the cerebral inhibitory GABAergic systems, a fact correlated by some authors with some symptoms of schizophrenia [141]. Oxidative stress produces mitochondrial dysfunction and also oligodendrocyte malfunction [142,143]. Oxidative stress is also involved in the myelination disorders present in schizophrenic patients [144]. Post-mortem studies showed a higher level of nitric oxide in the caudate nucleus of schizophrenia patients [145]. Zinc and magnesium both have an antioxidant action and reduce oxidative stress. Nuclear factor erythroid 2-related factor 2 (Nrf2) plays an important antioxidant role. Magnesium increases the activity of this factor. Magnesium isoglycyrrhizinate enhances the expression of Nrf2 and decreases free radical formation [106]. MgSO_4_ (600 mg/kg) protected mitochondria from neurons in conditions of increased oxidative stress [107]. Since some abnormalities in prenatal brain development and the influence of harmful factors including hypoxia are considered risk factors for the emergence of schizophrenia in postnatal life, the action of magnesium in reducing nuclear oxidative stress in the fetal brain is also important [146].

Zinc is a powerful antioxidant factor throughout the human body, including the brain. Zinc deficiency is associated with an increase in oxidative stress, increases in the plasma and tissue concentrations of F(2)-isoprostane (an important biomarker of oxidative stress) and a decrease in antioxidant defense mechanisms [147].

### 2.10. Mitochondria

There are various mitochondrial dysfunctions in schizophrenia, including mitochondrial hypoplasia, reductions in ATP synthesis and energy production and altered mitochondrial-related gene expression [148]. Zinc has an antiapoptotic action and preserves the mitochondrial integrity in neuronal aggression. A method of zinc mitochondrial protection is by inhibiting the apoptotic protein Lgals3 [149]. Mitochondria are very important for cellular energy production. Magnesium is essential for energy production and acts via Mg ATP complex formation. This cation is also important for the regulation of mitochondrial Ca^2+^ uptake. The decrease in concentration of magnesium [Mg^2+^] causes an increase in mitochondrial calcium uniporter (MCU) activity [150].

### 2.11. Some Active Endogenous Substances

#### 2.11.1. α.Norepinephrine

Norepinephrine (NE) is involved in the pathogenesis of schizophrenia and especially in the cognitive and motivational deficits present in schizophrenic patients. Existing data show that firstly the abnormal functioning of the NE system in the locus coeruleus (one of the regions with the highest concentrations of NE in the brain) is involved in these deficits. In addition to the higher level of NE, neurons in the locus coeruleus also release galanin [151]. There are studies that show that along with NMDA receptor hypofunction, the hyperactivity of NE systems is important in the pathogenesis of schizophrenia [152]. The level of NE is significantly higher in the brains of animals with a magnesium-deficient diet compared to those with a high magnesium diet [153]. Glutamate increases the release of NE by stimulating NMDA receptors. Magnesium reduces the release of this catecholamine in two ways: directly and by reducing the activity of NMDA receptors [154]. Zinc deficiency decreases the expression of the NE transporter (NET) in the locus coeruleus. This transporter is expressed in the presynaptic neuronal area and plays an important role in regulation of the NE synaptic concentration [155]. In experimental studies, animals fed a zinc-deficient diet have lower concentrations of NE in all brain regions compared to animals fed a normal diet [156]. Along with delirium and hallucinations, the cognitive and motivational deficits are essential elements in the symptomatology of the schizophrenic patient. There are strong interactions between NE, dopaminergic and glutamatergic systems in brain. Dopaminergic neurons have alpha1 and alpha2 receptors. NE has a modulatory role regarding the release of dopamine in the prefrontal cortex [151,157]. Magnesium increases N-acetyltransferase activity [158]. The concentrations and amounts of NE in the brains of rats that received a diet deficient in magnesium were significantly higher than in animals with a normal level of magnesium [159].

#### 2.11.2. β.Galanin

Galanin is a 30-amino-acid neuropeptide that is found in different regions of the brain and has multiple roles. By acting on the limbic system, it exhibits anxiolytic action [160]. Galanin inhibits the release of the most important neurotransmitters involved in the schizophrenia pathogenesis: dopamine, glutamate and serotonin [161]. The activity of neurons stimulated by NE in some brain regions is also influenced. This peptide inhibits the activity of noradrenergic neurons in the locus coeruleus. A higher concentration of magnesium and a lower concentration of calcium favor this action [162]. The inhibitory effect of galanin is produced by stimulating the galanin 1 receptor (GAL-R1). This receptor is not only found in the locus coeruleus but also in other regions of the brain. Polymorphisms of the 5′ region of the GALR1 have been identified in patients with schizophrenia [163]. There are data showing that galanin and zinc have a neuromodulating effect at the level of other G protein-coupled receptors.

In the brain, there are allosteric receptor–receptor interactions and the formation of homo- and heteroreceptor complexes. Galanin receptors are also involved in the formation of these complexes [164]. One such complex identified is the one between 5-HT1A receptors and GAL-1 receptors. The role of these complexes in the pathogenesis of schizophrenia and the importance of the GAL-1 receptor in these complexes is not yet known. Experimental studies in zinc-deficient Wistar rats showed that the activity of galanin decreases [165].

#### 2.11.3. γ.NF-kappaB

NF-kappa B factor is an important transcription factor involved in cytokine synthesis. The level of this factor is significantly higher in the cortex of schizophrenic patients compared to normal subjects [166]. A low level of dysbindin-1 (a regulatory protein regarding NF-kappa B factor activity) is also involved in schizophrenia pathogenesis. An increased degradation of this protein facilitates an increase NF-kappa B factor activity in the nucleus [167]. Magnesium deficiency causes the activation of NF-kappa B factor activation [168]. This is an important reason for magnesium administration in schizophrenia patients. A low level of Zn^2+^ also activates NF-kappaB factor [169].

#### 2.11.4. δ.Substance P (SP)

SP is a part of the tachykinin neuropeptide family. The role of this substance in schizophrenia is unclear. In schizophrenic patients, as well as in mood disorders subjects, the density of neurokinin-1 receptors in the cingulate cortex is unchanged compared to the normal control group [170].

Antagonists of D1 dopamine receptors reduce the level of SP [171]. In patients with chronic schizophrenia, haloperidol or risperidone administration decreased the SP level (29.0 ± 7.8 pg/mLin control subjects versus 20.6 ± 5.5 pg/mL in haloperidol-treated patients) [172]. The interactions between SP and magnesium are complex. Magnesium decreases the plasma concentration of SP. Some SP antagonists increased the concentration of intracellular magnesium in experimental studies [111]. A diet with a low magnesium content and deficiency in this cation increased the concentration of SP and also elevated the expression of SP receptors on the surfaces of T lymphocytes. Zinc has a modulating role in the release of SP from neurons. At low concentrations of zinc (10^−8^–10^−7^ M), neurons in cultures release an increased amount of this neuropeptide. At higher concentrations of zinc, the release of SP is greatly reduced or stops [173].

#### 2.11.5. ε.BDNF

BDNF is a very important factor for brain functions. This factor is essential in neural plasticity and also in neurogenesis and neuronal maturation. The plasma level of BDNF is significantly reduced in patients with a first episode of schizophrenia compared to healthy people [174]. As BDNF can cross the blood–brain barrier (BBB), it is considered that the plasma concentration reflects the situation in the brain [175]. BDNF reduces neuroinflammation and decreases the production of pro-inflammatory interleukins in the brain [176]. A reduction in BDNF is positively associated with the severity of schizophrenia and cognitive decline [109]. Magnesium increases the level of BDNF in the brain, especially in the hippocampus. A low-zinc diet in rats causes a decrease in BDNF concentration in the hippocampus [177].

#### 2.11.6. ζ.ACTH and Corticosteroids

Some studies have shown that the levels of ACTH and corticosteroids are significantly higher in drug-naïve patients with schizophrenia compared to normal control subjects (41.3 ± 14.6 vs. 12.4 ± 1.1 pg/mL, respectively; 279.4 ± 26.0 vs. 213.1 ± 18.4 nmol/L) during the first episode of the disease [178]. These data show the existence of important dysfunctions of the hypothalamic–pituitary–adrenal axis in schizophrenics, or at least in some of them. Other studies have demonstrated that stress increases the vulnerability to the onset of the first episode of schizophrenia. The levels of ACTH and cortisol in stress are high [179,180]. The administration of antipsychotics reduced the level of ACTH in schizophrenic patients [181]. Magnesium also decreases the synthesis of ACTH. Experimental studies in rats have shown that chronic administration of ACTH impairs long-term memory, while magnesium sulfate administered at 50 mg/kg/day for 4 weeks increases this memory [110]. We consider it a hypothesis that correcting hypomagnesemia in vulnerable people and the constant maintenance of a normal level of magnesium is crucial for preventing the occurrence of a first episode of schizophrenia, and also for reducing some of the memory disorders encountered in schizophrenic patients. A high level of corticosteroids enhances zinc elimination and decrease zinc plasma levels [182]. On the other hand, Zn^2+^ reduces cortisol secretion [183].

#### 2.11.7. η.Oxytocin

In the brain, oxytocin has various actions. It improves cognition, reduces aggression, increases memory and improves social behavior.

All these actions make it possible to involve the reduction in oxytocin activity in the etiopathogenesis of schizophrenia. Some authors recommend the use of oxytocin in the treatment of some patients with this disease [184] Oxytocin has the potential to improve the negative symptoms of schizophrenia. Mg^2+^ is important for the activation of oxytocin receptors by their natural agonist [185]. Normal levels of extra and intracellular magnesium increase the effects of oxytocin in the brain and can be beneficial for patients with schizophrenia. The formation of complexes with Zn^2+^ is an essential element for the action of oxytocin [186]. This cation also facilitates the activation of mitogen-activated protein kinase (MAPK) by oxytocin molecules, thereby amplifying the biological signal after the activation of oxytocin receptors [187].

#### 2.11.8. θ.Prolactin

Some antipsychotic drugs increase the prolactin level in schizophrenia patients. The role of this hormone in this disease’s evolution is debated. Mg^2+^ reduces prolactin secretion. Zn^2+^ also decreases prolactin synthesis and release from pituitary glands [188]. In normal people, zinc deficiency induces hyperprolactinemia [189]. The administration of zinc could be useful in reducing the prolactin plasma concentration in patients treated with antipsychotics. Since prolactin reduces the secretion of gonadal hormones, zinc could improve gonadal hormone secretion, leading to positive consequences for sexual behavior. In such cases, zinc can mitigate the side effects of some antipsychotics.

## 3. Some Important Processes Disturbed in Schizophrenia

### 3.1. Sleep

Schizophrenia is associated with important sleep disturbances [190]. Sleep abnormalities occur in 15–30% of patients with schizophrenia [191]. GABA deficiency plays a role in lack of sleep and in reducing the quality of sleep. Some authors claim that there is a deficit of GABA (B) receptor activity involved in these disturbances and that the administration of agonists of these receptors can have a favorable effect on sleep [191]. The reduced activity of GABA (B) receptors determines the hyperactivity of the dopaminergic systems strongly involved in the pathogenesis of schizophrenia. Decreased duration and quality of sleep are also involved in reducing memory because memory consolidation occurs in the second stage of sleep [192]. GABA (A) receptors also play a role in sleep disturbances in patients with schizophrenia because benzodiazepines increase the duration of sleep. Clinical studies on a large number of adult subjects have shown that magnesium administration is positively associated with an increase in sleep quality [193,194]. One of the mechanisms by which magnesium increases the quality and duration of sleep is the stimulation of melatonin secretion. In the case of magnesium deficiency, melatonin secretion decreases [195]. Regarding zinc, the data regarding the influence of this cation on sleep duration and quality are less clear and future clinical studies are necessary, but existing results indicate a positive effect of zinc on sleep quality [196].

### 3.2. Cognition

Cognition is reduced in different proportions in schizophrenia patients. Different forms of memory are affected in schizophrenia. One of the severely influenced ones is prospective memory [197]. This memory refers to the actions proposed to take place in the future. Patients with schizophrenia also have a decrease in immediate memory. Administration of magnesium threonate increased cognitive functions in healthy adults [198]. In experimental studies on animals, magnesium improved cognitive functions in damaged brains [199] and enhanced old rats’ memory and other cognitive functions and reduced anxiety [200,201]. Magnesium administration led to higher levels of hippocampus neurogenesis and neuroplasticity. Increases in the activity levels of TNF-α and NF-κB determine a decrease in memory and are also involved in the development of an emotional deficit. Emotional deficit manifests itself in human clinical practice through a deficit in facial recognition of emotion, attention deficits and other symptoms [202].

Magnesium reduces the activity of these two factors, while a deficit of this cation increases it [203]. Another way magnesium can stimulate memory and prevent its decline is through the action on the NR2B subunit of NMDA receptors. Decreased activity of this NMDA receptor subunit is associated with decreased synaptic plasticity and memory decline. Magnesium can boost the NR2B subunits’ activity and alleviate or at least partially prevent memory decline [204].

Zinc is involved in cognition. In all situations, zinc deficiency leads to impaired cognition [205]. Intracellular zinc is important for the activation of the NF-kappaB factor. This factor is involved in several deficient processes in persons with schizophrenia, including cognition [206]. Cognitive processes are affected especially by zinc deficiency in the hippocampus [63].

The implications of zinc in the schizophrenia pathogenesis are shown in Table 2.

### 3.3. Gut Microbiome

The gut microbiome is a very important ecosystem, not only for the functioning of the digestive system but also for the brain. A number of compounds produced by bacteria at the intestinal level reach the brain through the gut–brain axis and influence the brain’s neurotransmitter and neuromodulator systems [207,208]. The disorders that appear at the level of the gut microbiome are associated not only with digestive diseases but also with other diseases, including psychiatric diseases such as schizophrenia [209]. A higher production rate of serotonin by the gut microbiome was positively correlated with an increased risk of schizophrenia [205,210]. Zn^2+^ is very important not only for the human body but also for some bacteria in the intestinal microbiome. The deficiency of this cation alters this microbiome [211,212].

Dysfunctions of the glutamatergic and GABAergic systems are very important in the pathogenesis of schizophrenia. The gut microbiome influences the functioning of these systems. The *Lactobacillaceae* family is important within the normal intestinal microbiome. *Lactobacillaceae* have zinc transporters [213]. The absence of Zn^2+^ greatly affects this family of intestinal bacteria. This biometal is also important for the normal functioning of the intestinal barrier. Dysfunctions of this barrier allow metabolites to reach the brain that can disrupt the functioning of the brain’s neurotransmitter systems.

Magnesium deficiency affects the intestinal microbiome. Experimental studies on rodents have shown that six weeks of a magnesium-deficient diet are sufficient to produce important changes in the structure of the intestinal microbiome [214]. The administration of magnesium has a modulatory role on the intestinal microbiome, increases the population of *Bifidobacterium* and has an effect of reducing intestinal inflammation [215].

Anxiety is frequently encountered in patients with schizophrenia. This reduction in anxiety occurred simultaneously with the reduction in intestinal dysbiosis and with the decrease in the population of commensal sulfate-reducing bacteria [216]. In this way, magnesium could be involved in reducing anxiety in schizophrenia patients.

### 3.4. Appetite

Overweight and obesity are frequently encountered in patients with schizophrenia. Between 40% and 62% of people with schizophrenia are obese or overweight.

Appetite disorders are also common in patients with schizophrenia. Therapy with some antipsychotics such as olanzapine, risperidone and others causes weight gain and sometimes obesity [217]. Zinc is an appetite stimulant. An essential mechanism for the increase in appetite by zinc is the reduction in gene expression of hypothalamic ghrelin [218].

The increase in zinc concentration after treatment with some antipsychotics can be a factor in the increase in appetite in these patients with schizophrenia. On the other hand, hypomagnesemia is involved in the obesity encountered in quite frequent cases of schizophrenia. The administration of magnesium (450 mg/day 6–24 weeks) led to a reduction in Body Mass Index (BMI) in obese adults [219].

## 4. Genetics

Schizophrenia is a polygenic disease. At least 108 genes are more or less associated with an increased risk of this disease [220]. A direct action of zinc or magnesium on these genes is not known, but among the many zinc-dependent enzymes are RNA polymerase and DNA polymerase, with important roles in the formation of nucleic acids. Some Zn-binding proteins also act as DNA-binding transcription factors [118].

## 5. Therapeutics

At the therapeutic level, an important number of antipsychotic drugs used in the human clinic act at the level of serotonergic receptors. There are preliminary studies that show that the administration of magnesium increases the therapeutic effect of some antipsychotics (inhibitors of serotonin re-uptake) [221]. Administration of zinc (220 mg zinc sulfate/day per os) for six weeks significantly reduced both positive and negative symptoms in patients with schizophrenia undergoing atypical antipsychotic pharmacotherapy [222]. A double-blind, randomized, placebo-controlled trial showed that the administration of zinc sulfate (50 mg elemental zinc/day per os) in combination with risperidone (6 mg/day) in patients with schizophrenia led to a significantly greater improvement in the condition of the schizophrenic patients (assessed with the Positive and Negative Syndrome Scale (PNSS)) compared to the group that received only risperidone [223].

There are data that show that during the clinical remission of patients with schizophrenia, the plasma magnesium level increases [16]. Other studies showed increases in the plasma concentrations of magnesium both after treatment with classic antipsychotics and with atypical antipsychotics [224].

The influence of various antipsychotics used in the therapy of schizophrenia on magnesium and zinc concentrations is controversial but the number of clinical studies in this direction is relatively small. Haloperidol increases the plasma level of magnesium [225]. In another clinical study, a significant increase in serum magnesium was reported after a few weeks of treatment with antipsychotics, but not an increase in zinc plasma levels [226]. Some studies showed significant increases in plasma zinc and erythrocyte magnesium concentrations and a decrease in copper concentration after treatment with olanzapine at 6 mg/day and haloperidol for several weeks [227].

Motility disorders and cataleptic states are sometimes encountered in patients with schizophrenia. Some typical classical antipsychotics such as haloperidol, through their action on the extrapyramidal system, cause adverse effects such as catalepsy, tardive dyskinesia and orofacial disfunctions. These drugs can also aggravate Parkinson’s disease.

Catalepsy is produced by blocking D1 and D2 receptors. The administration of magnesium simultaneously with haloperidol prevented the occurrence of these adverse effects, and the administration of this biometal (6–28 days) after the onset of extrapyramidal disorders reduced their intensity [227]. Experimental studies have shown that catalepsy induced by classic antipsychotics in mice is prevented by zinc administration at 15 mg/kg/day [228].

## 6. Nutrition

A clear evaluation of the roles of magnesium and zinc in nutrition in the development of schizophrenia and a correlation between the amounts of zinc and magnesium introduced into the body at different ages and the occurrence of schizophrenia in some people is missing. Various studies have shown that a reduced nutritional intake of zinc is associated with an increased risk of schizophrenia [229], but it is not clear how long this deficit must be for the risk of this disease to be significantly higher. Zinc is also important in early brain development. The production of growth factors and the development of brain synapses is dependent on zinc intake [230]. Some studies show that in schizophrenics aged between 18 and 65 years, the nutritional intake of magnesium is lower than in normal people [231,232]. Amongst nutraceuticals, the World Federation of Societies of Biological Psychiatry (WFSBP) recommends zinc for patients with schizophrenia [233].

## 7. Conclusions

The implications of magnesium and zinc in the pathogenesis of schizophrenia are complex and only partially known. There are both clinical and experimental data that support this conclusion. The data obtained using experimental models of schizophrenia in animals must be interpreted and translated into clinical practice with caution. An experimental model that accurately reproduces schizophrenia in animals does not exist. However, only clinical and experimental data interpreted, analyzed and correlated together open the way to understanding the pathogenic mechanisms of schizophrenia. Although there are no data regarding these implications in all types of schizophrenia, because in all clinical studies zinc levels are significantly low in these patients and magnesium levels are low in many patients, the magnesium and zinc deficits must be corrected. A normal level of these cations is definitely beneficial for patients with this disease. There are clinical data that show that the association of the two cations with some antipsychotic drugs is useful for a better therapeutic effect, as well as clinical results that show that the administration of some antipsychotic drugs increases intracellular zinc and magnesium levels. We hypothesized only that this increase is part of the mechanism of action of these antipsychotic drugs. Experimental studies have shown that some of the actions of the two cations reduce the known molecular mechanisms of schizophrenia.

It is important to correct the cation deficits as early as possible after the diagnosis of the disease and to take into account the possibility that some drugs administered for other diseases associated with schizophrenia increase the elimination of the two cations from the body. The daily dose of zinc should not exceed 25 mg. An excess of the recommended daily dose may result in symptoms such as anemia, neutropenia and decreased plasma and tissue copper concentrations. The daily dose of magnesium administered orally to patients with hypomagnesemia should not exceed 400 mg.

In the future, clinical studies regarding the importance of the concentrations of zinc and magnesium for reducing the number of relapses of the disease are needed.

Considering the heterogeneity of the disease, studies are needed for all types of schizophrenia and for different ages of patients. It is very important that future studies show whether patients were diagnosed with low levels of zinc and magnesium before the first clinical manifestations of schizophrenia appeared. Another research direction should follow whether the long-term results of antipsychotic therapy are influenced by the concentrations of the two cations.

We considered only a hypothesis that normal levels of these cations could prevent the onset of the disease in some cases and could prevent relapses in other cases.

## Figures and Tables

**Table 1:** The implications of magnesium in the pathogenesis of schizophrenia.

	Mg^2+^ Actions
**Dopaminergic system**	-Magnesium at a dose of 1.2 mM reduced, in experimental studies, the NMDA-induced release of dopamine in the striatum [48,49]. -Mg^2+^ reduces striatal dopamine release stimulated by Ca^2+^. -Mg^2+^ decreases striatal dopamine release stimulated by stress [15]. -Absence of Mg^2+^ in the superfusion buffer in microdialysis studies resulted in doubling of basal dopamine release [46].
**Glutamatergic system**	-Mg^2+^ blocks the calcium channels coupled to NMDA receptors [67,68]. -Mg^2+^ reduces glutamate-induced dopamine release [68].
**GABA-minergic system**	-In experimental studies, at a concentration of 10 mM of magnesium, the binding of GABA receptor agonists to GABA receptors (A) was increased [84]. -Mg^2+^anxiolytic effect is partially mediated by GABA-A receptors activity [85]. -In cell culture, Mg^2+^ stimulates GABA neuron development [87]. -Mg^2+^ increases CREB and mTOR neuronal activity [88].
**Serotoninergic system**	-MgCl_2_ reduces 5-HT concentration in Nc. accumbens and prefrontal areas [78].
**Cannabinoid system**	-Mg^2+^ decreases THC neurotoxicity [94]. -In experimental studies, THC inhibits Mg-ATP-ase activity [95].
**Cholinergic system**	-Mg^2+^ blocks Na^+^ channels coupled with nicotinic receptors [101]. -Mg^2+^ (1.2–9.3 mM) increases the acetylcholine release from rat cortex slices [105].
**Oxidative stress**	-Mg^2+^ increases Nrf2 expression [106]. -Mg^2+^ decreases free radical formation [106]. -Mg^2+^ protects the neuronal mitochondria in oxidative stress conditions [107].
**Neuroinflammation**	-Mg^2+^ decreases the synthesis of proinflammatory cytokines [108].
**Neuroplasticity**	-Mg^2+^ reduces neuronal apoptosis and stimulates brain stem cell neurogenesis [5]. -Reduction in the BDNF level is positively associated with the severity of schizophrenia and cognitive decline, but Mg^2+^ increases the BDNF level in the hippocampus and also neuroplasticity [109].
**ACTH and corticosteroids**	-In experimental studies in rats, Mg^2+^ decreases ACTH and corticosteroid synthesis [110].
**Substance P**	-In a diet with a low magnesium content, Mg^2+^ decreased substance P concentration and also elevated expression of SP receptors on the surface of T lymphocytes [111].

**Table 2:** Zinc’s implications in schizophrenia pathogenesis.

	Zn^2+^ Actions
**Dopaminergic system**	-Zn^2+^ reduces dopamine transport and regulates dopamine transporter activity [41]. -In experimental studies on rats, Zn^2+^ increased dopamine concentrations in some brain areas [42].
**Glutamatergic system**	-Zn^2+^ is an allosteric antagonist of NMDA receptors [66]. -Experimental studies have shown that low Zn^2+^ concentrations potentiate AMPA receptor activity [59,60]. -Zinc reduces the increases in extracellular glutamate concentration produced by some factors [63].
**GABA-minergic system**	-Zn^2+^ binds to a subunit of GABA-A receptors. -In zinc-deprived rats, the extracellular concentration of GABA decreases [89].
**Serotoninergic system**	-Zn^2+^ is an allosteric modulator of 5-HT(1A) receptors and reduces the binding of agonists to 5-HT(1A)receptors [78]
**Cholinergic system**	-Zn^2+^ potentiates the activity of alpha4 and beta 4 nAch receptors [112]. -Zn^2+^ increases the currents elicited by Ach at the level of nAch receptors [112].
**Mitochondria**	-Experimental studies have shown that Zn^2+^ inhibits the activity of some proapoptotic proteins, and this cation preserves the mitochondria integrity [149].
**Different endogenous substances**	-Experimental studies in zinc-deficient Wistar rats showed that the activity of galanin decreases [165]. -Zn^2+^decreases NF-kB factor activity [169]. -At low concentrations of zinc (10^−8^–10^−7^ M), neurons in cultures release an increased amount of SP [173]. -Zn^2+^ reduces cortisol secretion [183]. -A high level of corticosteroids enhances zinc elimination and decreases zinc plasma levels [182]. -Zn^2+^ facilitates the activation of MAPK by oxytocin [187]. -In experimental studies, Zn^2+^ reduces prolactin secretion [188]. -In normal people, zinc deficiency induces hyperprolactinemia [189].
**Neuroinflammation**	-Zn^2+^ inhibits the synthesis of proinflammatory cytokines [108]. -Zn^2+^ decreases oxidative stress [147]. -In experimental studies in rats, Zn^2+^reduced F2-isoprostane synthesis [147].
**Neuroplasticity**	-Zn^2+^ reduces apoptosis and increases neuroplasticity. -Zn^2+^ deficiency inhibits cell proliferation. In cell cultures, with a low level of zinc, the cell cycle stops in the G0/G1 phase [118].
